# Antagonism of stress granules is key to SARS-CoV-2 infection and pathogenesis

**DOI:** 10.1128/jvi.00992-26

**Published:** 2026-09-08

**Authors:** R. Elias Alvarado, Jennifer Chen, Kumari G. Lokugamage, Yiyang Zhou, Leah K. Estes, Angelica L. Morgan, Yani Ahearn, Xiangxue Deng, Lilin Lai, Arian Moayyed, William Meyers, Jessica A. Plante, Ken S. Plante, David H. Walker, Xuping Xie, Mehul S. Suthar, Bryan A. Johnson, Vineet D. Menachery

**Affiliations:** 1 Department of Microbiology & Immunology, University of Texas Medical Branch 12338 , Galveston, Texas, USA; 2 Human Pathophysiology and Translational Medicine, University of Texas Medical Branch 12338 , Galveston, Texas, USA; 3 Institute for Translational Sciences, University of Texas Medical Branch 12338 , Galveston, Texas, USA; 4 Department of Pediatrics, Emory University School of Medicine 12239 , Atlanta, Georgia, USA; 5 Emory Vaccine Center, Emory University 1371 , Atlanta, Georgia, USA; 6 World Reference Center of Emerging Viruses and Arboviruses, University of Texas Medical Branch 12338 , Galveston, Texas, USA; 7 Department of Pathology, University of Texas Medical Branch 12338 , Galveston, Texas, USA; 8 Center for Biodefense & Emerging Infectious Diseases, University of Texas Medical Branch 12338 , Galveston, Texas, USA; 9 Institute for Human Infection and Immunity, University of Texas Medical Branch 12338 , Galveston, Texas, USA; University of Minnesota Twin Cities, Minneapolis, Minnesota, USA

**Keywords:** SARS-CoV-2, coronavirus, NSP3, FXR1, nucleocapsid, stress granules, G3BP1

## Abstract

Viruses must subvert host responses to facilitate successful infection. While most antiviral responses are associated with interferons, stress granules (SGs) are another barrier to viral infection by inducing translation arrest. To combat SG activity, viruses have evolved mechanisms to disrupt formation and disassemble these complexes. Our prior studies identified residues in SARS-CoV-2 NSP3 (Y138/F145) and nucleocapsid (F17) that independently antagonize SG activity. Disrupting these key residues in NSP3 or nucleocapsid attenuated viral replication, but only modestly impacted *in vivo* pathogenesis, suggesting overlap in SG antagonism partially compensates for the individual losses. In this study, we evaluated a SARS-CoV-2 mutant (YF/F17A) that combines the NSP3 and N mutations. We find that loss of both SG-antagonizing functions attenuates SARS-CoV-2 replication *in vitro*. While no changes are seen in type I interferon sensitivity, attenuation corresponds to increased induction of SGs. Importantly, the SARS-CoV-2 YF/F17A mutant attenuates significantly *in vivo,* with reduced viral replication, less weight loss, and limited immune pathology. Notably, infection with the YF/F17A mutant stimulated less interferon and inflammation. Despite these muted host responses, the YF/F17A mutant stimulated robust protection against subsequent challenge with WT SARS-CoV-2. Overall, the study highlights the importance of SG control for SARS-CoV-2 infection and offers a novel, interferon-independent target for vaccination and therapeutic treatment going forward.

**IMPORTANCE:**

This study demonstrates that SARS-CoV-2 uses multiple mechanisms to block host stress granules during infection. Knocking out both N- and NSP3-mediated antagonism of stress granules attenuates viral replication and disease caused by SARS-CoV-2. Importantly, while most therapeutics target key viral processes or induce interferon pathways, this study shows stress granule activation as a novel approach to attenuate and treat coronavirus infection.

## INTRODUCTION

Coronaviruses are positive-sense, enveloped RNA viruses that have posed significant global threats to human health and led to three pandemics this century (1–3). To establish infection, viruses must interact with host cellular components to establish infection and generate progeny virus (4). Therefore, understanding these host–virus interactions is paramount for developing new antivirals and vaccine candidates.

Eukaryotic cells have evolved defense mechanisms to sense and respond to cellular stresses, including viral infections (5–7). When cells encounter stress, they rapidly arrest translation by activating stress-responsive kinases that disrupt formation of the eukaryotic translation initiation factor 4F (eIF4F) complex (8). Accumulated untranslated mRNAs engage with host proteins to form cytosolic foci termed stress granules (SGs). SGs are transient ribonucleoprotein (RNP) condensates formed via liquid–liquid phase separation, in which stalled translation preinitiation complexes, cellular mRNAs, small ribosomal subunits, and RNA-binding proteins such as Ras GTPase-activation protein-binding 1 (G3BP1) and Fragile X mental retardation proteins (FMRPs) coalesce into a concentrated phase (9). Host cells respond to stress such as viral infections by forming SGs that can further disrupt host translation by sequestering these key factors. To counteract SGs, some viruses respond by hijacking, delaying, or disrupting SG formation during infection to ensure host machinery maintains translation of viral proteins (10–12). Taken together, SGs are a critical obstacle for viruses to overcome during infection.

Prior studies have shown that SARS-CoV-2 inhibits SG formation through multiple virus–host protein interactions (13–20). The nucleocapsid (N) protein of SARS-CoV-2 is a key structural protein that has several roles during infection and is primarily known for binding viral RNA and facilitating its incorporation into new virions (21). N also suppresses innate immune responses and supports viral RNA replication and transcription (22–24). These different roles for N can be regulated by kinetics, viral RNA regulation, and protein phosphorylation (25–27). Recently, our lab and others have shown that residue F17 in the N protein binds the G3BP1 NTF2L domain with high affinity and promotes SG disassembly (13, 28, 29). Interestingly, we found that mutating residue F17 to alanine (F17A) in a SARS-CoV-2 mutant resulted in persistent SGs at late time points (24 h post-infection [HPI]) (13). These observations indicate that N is a strong antagonist of SGs by inhibiting their functions at late times during infection.

In addition to N, SARS-CoV-2 nonstructural protein 3 (NSP3) can also inhibit SG formation. Our group identified that the hyper-variable region (HVR) of NSP3 binds the SG-related Fragile X mental retardation protein 1 (FXR1) via interaction with ubiquitin-associated protein 2-like (UBA2PL) (30). Disruption of the HVR region via alanine scanning resulted in enhanced SG formation and attenuated viral infection (6 HPI) (30). We subsequently identified two key aromatic residues in the NSP3 HVR, tyrosine (Y) 138 and phenylalanine (F) 145, responsible for binding UBA2PL (14). A SARS-CoV-2 mutant disrupting these residues (YF) had SG formation at early times (6 HPI) that was subsequently inhibited at later stages of infection (10 HPI). The disruption of SGs at late times corresponds to increased N levels at late times during infection. Together, our results from two separate studies demonstrate that NSP3 and N selectively target SGs at different times during infection (14). With such a significant investment in genetic capital, these results suggest control of SG formation is paramount to SARS-CoV-2 infection and pathogenesis.

To explore this idea, we examined the impact of SG activation by generating recombinant SARS-CoV-2 bearing mutations in the NSP3-HVR and N (YF/F17A) that prevent binding to FXR1 and G3BP1, respectively. We demonstrate that the SARS-CoV-2 YF/F17A mutant has reduced viral replication *in vitro,* which corresponded to increased SG formation rather than type I interferon (IFN) activation. We also observed attenuated pathogenesis *in vivo* with reduced disease and host responses. Notably, we show that despite diminished immune activation during primary infection, the YF/F17A mutant conferred protective immunity following vaccination and subsequent rechallenge with WT SARS-CoV-2. Overall, our studies demonstrate the importance of controlling SGs for SARS-CoV-2 infection and pathogenesis. Similarly, these findings demonstrate SG activation as a viable strategy for vaccination and therapeutic development for coronavirus infection.

## RESULTS

### Disruption of NSP3 and N stress granule antagonism attenuates SARS-CoV-2 infection

Our prior work identified two distinct mechanisms that disrupt SG formation during SARS-CoV-2 infection: (i) F17A mutation in SARS-CoV-2 N, which disrupts binding to G3BP1/2 (13) and (ii) Y138A/F145A (YF) in SARS-CoV-2 NSP3, which disrupts binding to FMRPs (14, 30). Although both mutants are moderately attenuated, their targeting of distinct SG components and differential effects on the temporal kinetics indicate that they disrupt two independent mechanisms of SG activation (Fig. 1a) (13, 14, 30). Consequently, we concluded that mutation at both interfaces may fully restore SGs during SARS-CoV-2 infection. Using our reverse genetics system (31, 32), we generated an NSP3/N double mutant (YF/F17A) in the SARS-CoV-2 WA-1 strain backbone (33, 34) (Fig. 1b).

**Fig 1:**
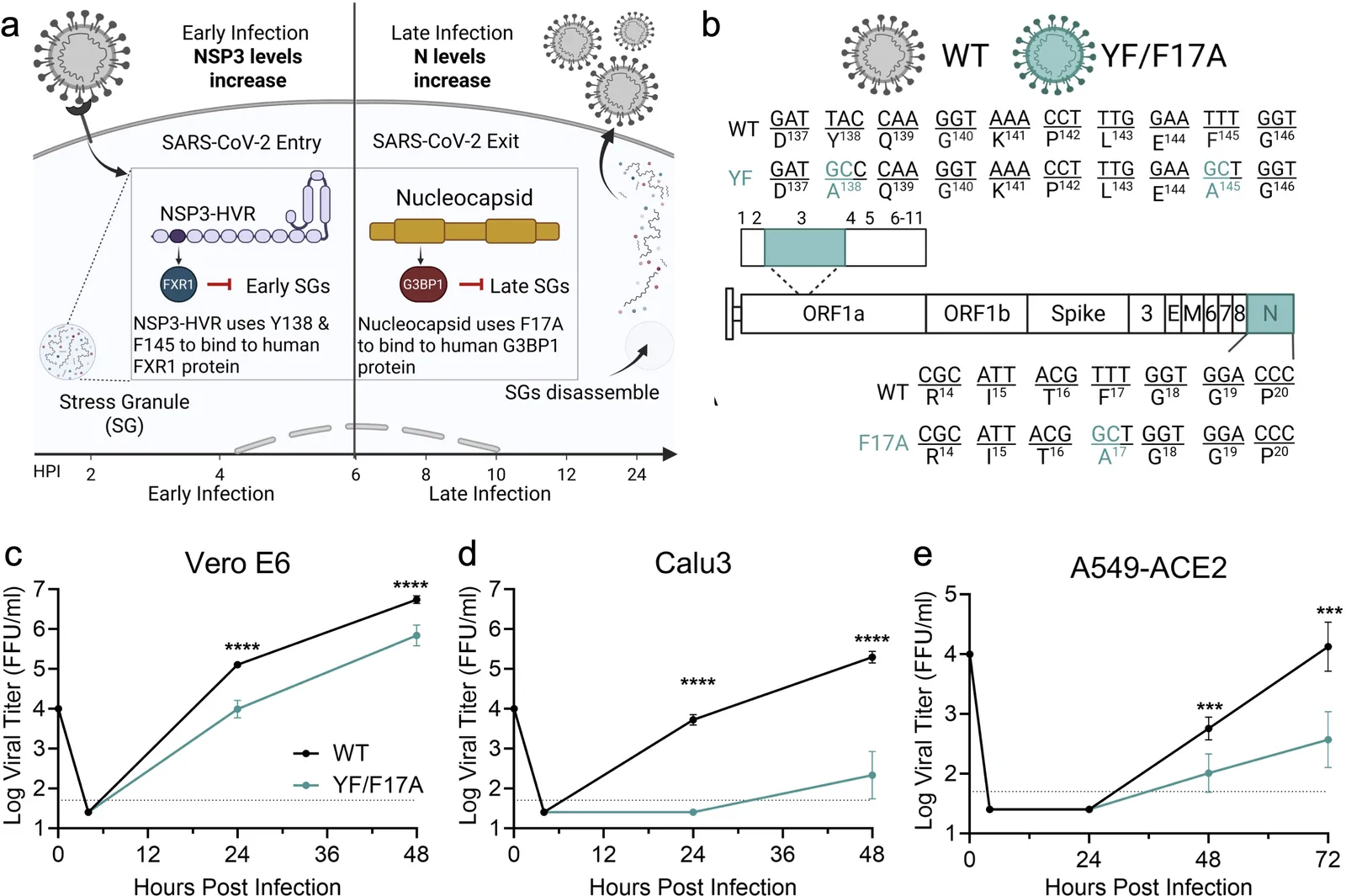
SARS-CoV-2 YF/F17A mutant has attenuated viral replication *in vitro*. (**a**) Proposed model of SARS-CoV-2 SG regulation by NSP3 and N protein at early and late stages of infection, respectively. (**b**) Schematic of SARS-CoV-2 double mutant (YF/F17A) with NSP3-YF and N-F17 mutations to alanine (teal) introduced in Washington 1 (WA-1) strain. (**c–e**) Viral titers in Vero E6 (**c**), Calu3 (**d**), or A549-ACE2 (**e**) cells infected with WT (black) or YF/F17A (teal) SARS-CoV-2 at an MOI of 0.01 (*n* = 6 from two experiments, each with three biological replicates). Data are representative of the mean ± SD. Statistical analysis was performed using two-tailed Student’s *t*-test. ***P ≤ 0.001; ****P<0.0001. Panels a and b were created in BioRender. Menacherylab, T. (2026) https://BioRender.com/wwctzhe, https://BioRender.com/3l1uj0i.

We first evaluated the viral replication of YF/F17A compared with WT SARS-CoV-2 in cell culture using a low multiplicity of infection (MOI of 0.01). Three cell models were selected for this analysis: Vero E6, an IFN-deficient cell line (35, 36); Calu3, a human bronchial cell line with intact interferon IFN responses (34, 37, 38); and A549-ACE2 cells, a lung adenocarcinoma cell line expressing human ACE2, with intact IFN responses and known stable SG activation (37, 39). Vero E6 growth curves revealed significant attenuation between the WT and YF/F17A at 24 and 48 HPI (Fig. 1c). In Calu3 cells, YF/F17A mutant virus was not detected at 24 HPI for YF/F17A mutant, while WT SARS-CoV-2 grew to 10^4^ FFU/mL (Fig. 1d); by 48 HPI, YF/F17A was observed, but was attenuated 3-log compared to control (Fig. 1d). In A549-ACE2 cells, the YF/F17A mutant replication was similarly attenuated relative to WT SARS-CoV-2 at 48 and 72 HPI (Fig. 1e). Collectively, these data show that the loss of SG antagonism via the mutation of both the NSP3-FXR1 and N-G3BP1/2 interaction mechanisms strongly attenuates SARS-CoV-2 replication *in vitro*.

### YF/F17A mutant attenuation is not primarily due to type I interferon responses

Both NSP3 and N are multi-functional proteins in the context of CoV infection with roles in viral RNA and protein regulation, host–virus interactions, and modulation of host immune responses (40). Given the level of attenuation of the YF/F17A mutant, the combined disruption may unexpectedly impact type I IFN responses. While neither the YF nor F17A individual mutants induce significant changes in innate immune responses (13, 14, 30), we evaluated YF/F17A replication for IFN sensitivity on Vero E6 cells, which lack the capacity to produce type I IFN, but respond to exogenous treatment (36). Pretreatment with 100 U of recombinant type I IFN (IFN-α) prior to infection, WT SARS-CoV-2 displayed modest sensitivity to type I IFN pretreatment with ~1-log reduction in viral titers at 24 HPI (Fig. 2a). Similarly, the YF/F17A mutant also displayed only modest IFN sensitivity with a ~1-log reduction in viral titer at 24 HPI (Fig. 2a). Unlike IFN-sensitive SARS-CoV-2 mutants that often display >3-log attenuation after pretreatment (41), these data are consistent with our prior studies with the YF and F17A individual mutants and argue that type I IFN signaling is not the predominant cause of the YF/F17A mutant attenuation (14).

**Fig 2:**
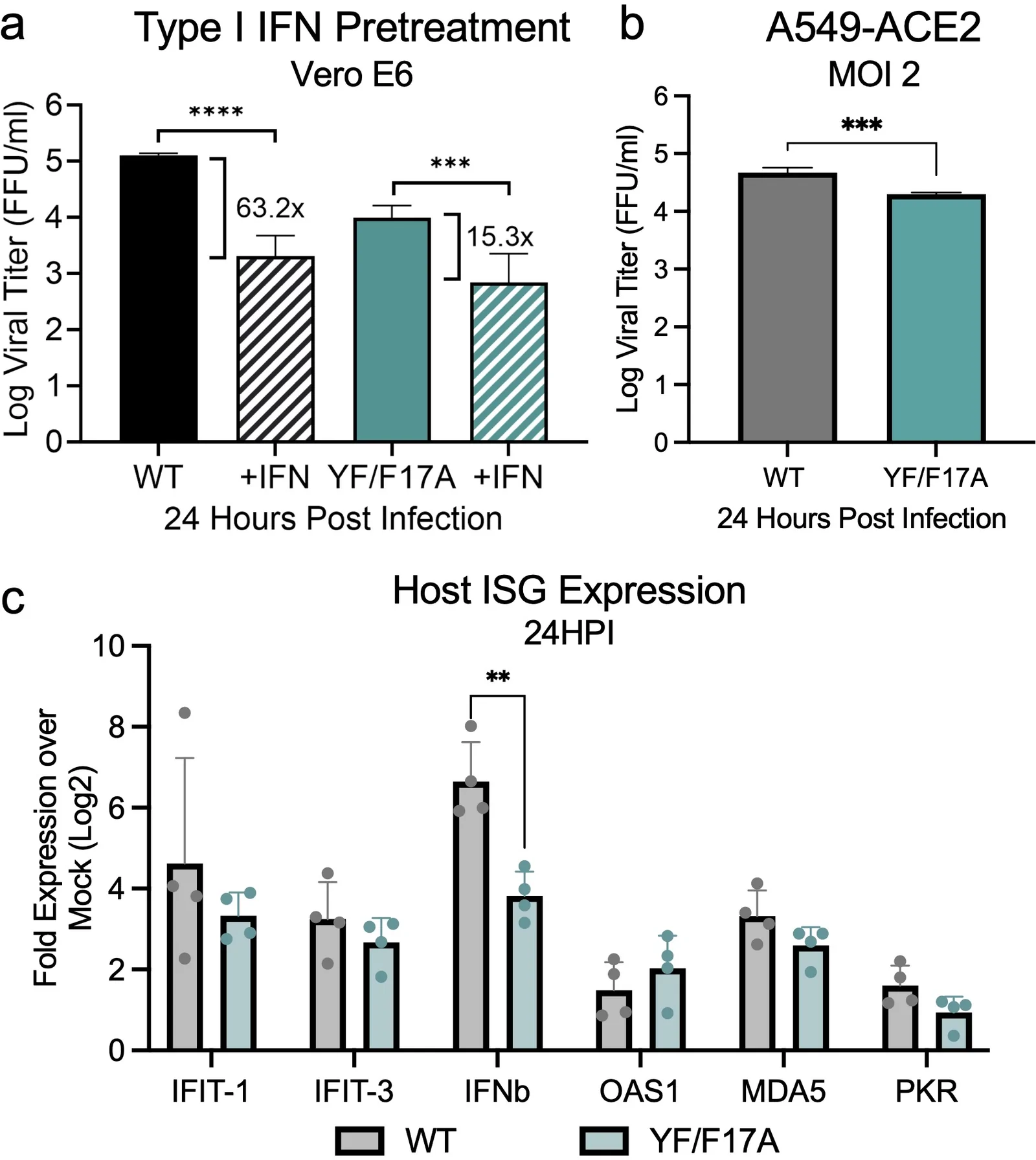
Decreased YF/F17A mutant replication is not driven by innate immune responses. (**a**) Vero E6 cells were pretreated with 100 U of recombinant type 1 IFN (hashed bar) or mock (solid bar) for 16 h prior to infection. Cells were subsequently infected with either SARS-CoV-2 WT (black) or YF/F17A mutant (teal) at an MOI of 0.01. Viral titers were measured at 24 HPI. The fold change relative to the mock control is shown. (**b and c**) A549-ACE2 cells were mock, WT, or YF/F17A infected at an MOI of 1 for 24 h, with viral titer shown at the endpoint. (**c**) At 24 HPI, total RNA was collected, and host mRNA expression was quantified by RT-quantitative PCR (qPCR). Ct values were normalized to human GAPDH and expressed as fold change (log_2_) of expression of indicated genes. Each dot represents a replicate, with means for each target displayed, ±SD (error bars). Statistical analysis was performed using two-tailed Student’s *t*-test. **P ≤ 0.01; ***P ≤ 0.001; ****P < 0.0001.

Although the YF/F17A mutant is not more sensitive to type I IFN pretreatment, it is possible that infection may induce augmented type I IFN responses resulting in reduced capacity to infect bystander cells. To explore this possibility, we infected human A549 cells expressing hACE2 with WT and YF/F17A at a high MOI (2). Following infection, we found that both WT and YF/F17A had similar titers at 24 HPI (Fig. 2b). Examining host RNA expression, we found few changes in a panel of type I interferon (*IFNB1*) and interferon-stimulated genes (ISGs; *IFIT1, IFIT3, OAS1, MDA5,* and *PKR*), consistent with previous work (Fig. 2c) (14). Most ISGs maintained similar levels of expression between WT and YF/F17A mutant-infected cells. However, IFNβ levels were augmented in WT as compared to the YF/F17A mutant. Together, the ISG and IFN gene expression data indicate that the YF/F17A mutant does not increase host ISG responses. Overall, these data are consistent with prior studies and confirm that attenuation of the YF/F17A mutant is not due to changes or increases in host innate immune responses.

### Reduced YF/F17A mutant replication corresponds to high levels of stress granule formation

Our prior studies have indicated that loss of either NSP3 interaction with FMRP proteins or N interaction with G3BP1 increases SG activation (13, 30). Here, we evaluated induction of SGs in the YF/F17A mutant following infection of A549-ACE2 cells. Briefly, A549-ACE2 cells were plated and infected with WT SARS-CoV-2, the YF/F17A mutant, or mock-infected. At 6, 10, and 24 HPI, cells were fixed, permeabilized, stained, and evaluated for SG formation by examining the number of cells staining positive for both SARS-CoV-2 N (red) and host G3BP1 (green) foci. Consistent with prior studies (13, 14, 30), WT SARS-CoV-2 fails to induce SGs at any time point following infection, while the YF/F17A mutant has strong induction comparatively (Fig. 3). At 6 HPI, SG formation is not observed in mock, WT, or YF/F17A mutant (Fig. 3a through d). While N protein should be produced by this time point, very few cells in either infected group are positive, suggesting limitations in staining sensitivity. By 10 HPI, WT SARS-CoV-2 still has minimal SG formation, similar to mock, as measured by G3BP1 foci, but now accompanied by significant N staining (Fig. 3e and f). In contrast, we observed a major increase in cells with both G3BP1 foci and N staining in the YF/F17A mutant (Fig. 3g and h). While not all N-positive cells had G3BP1 foci, a significant portion indicated the loss of SG control in the YF/F17A mutant. The loss of SG control in the YF/F17A mutant is maintained at 24 HPI, as over 1/4 of cells show evidence of SG induction compared to <3% in the WT-infected group (Fig. 3i through l). Together, these results demonstrate that the loss of both NSP3 and N antagonism allows viral infection to induce a significant level of SGs.

**Fig 3:**
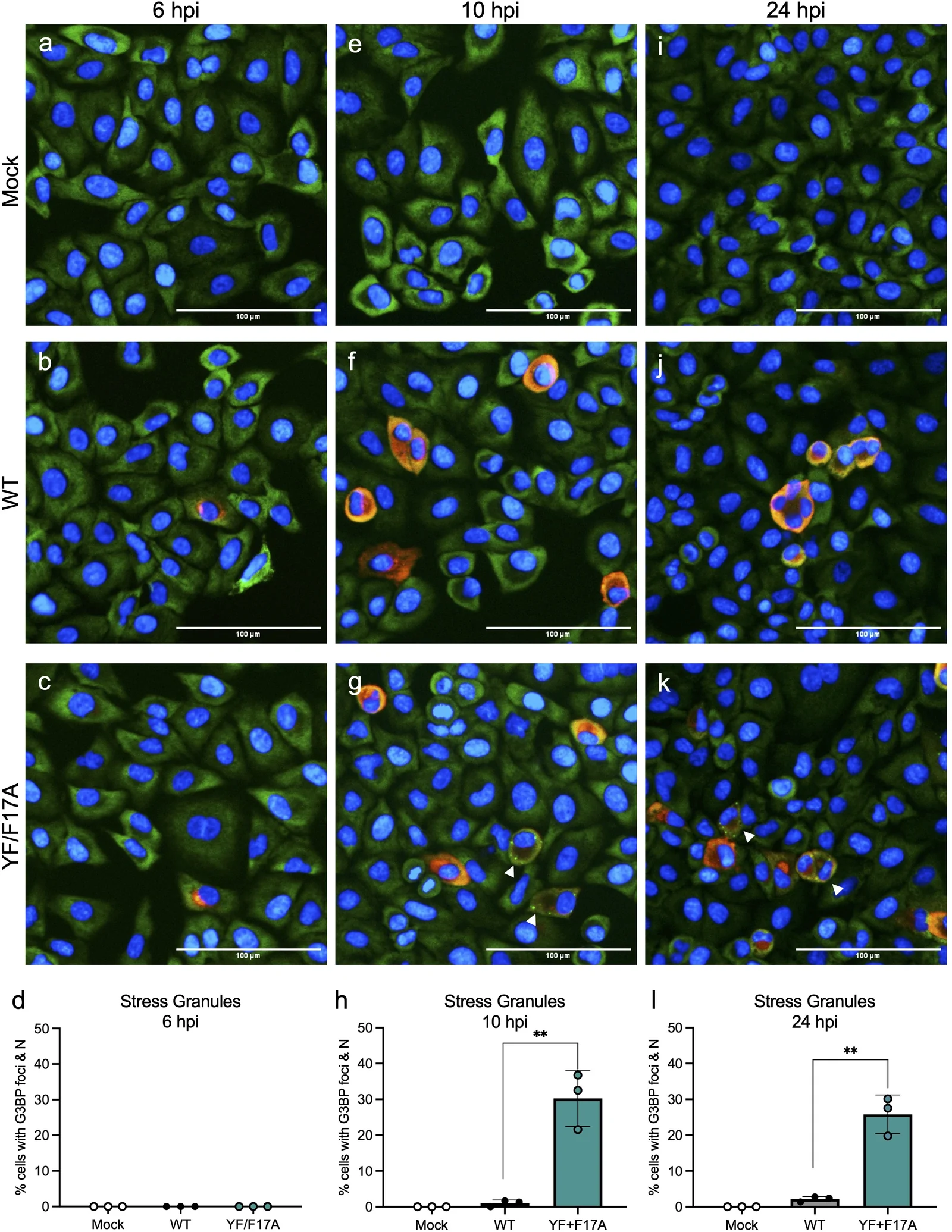
Stress granule formation is observed in the YF/F17A mutant. (**a–l**) A549-ACE2 cells were infected with mock (phosphate-buffered saline [PBS]), WT, or YF/F17A mutant at an MOI of 2 and visualized and quantified for SG formation at (**a–d**) 6 HPI, (**e–h**) 10 HPI, and (**i–l**) 24 HPI. G3BP1 (green), nucleocapsid (red), and nuclei (blue) were labeled to visualize by immunofluorescence imaging. The total percentage of cells with G3BP1+ foci at 6, 10, and 24 HPI was calculated. All quantitative data were calculated using Fiji (ImageJ) software and are shown as mean ± SD. Statistical analysis was performed using a two-tailed Student’s *t*-test. **P ≤ 0.01.

### YF/F17A mutant is unable to reverse arsenite-induced SGs

While SG antagonism is absent in the YF/F17A mutant, SARS-CoV-2 maintains numerous other viral antagonists to limit activation by double-stranded RNA. To further evaluate SG activation and control, we examined infection after sodium arsenite treatment, a known inducer of SGs (42).

Briefly, A549-ACE2 cells were infected with mock (PBS), SARS-CoV-2 WT, or YF/F17A mutant as described above; 30 min prior to the infection time point, sodium arsenite-containing media was used to replace culture media followed by inactivation and staining (Fig. 4). Consistent with prior studies, treatment with arsenite induced formation of SGs in mock-infected cells at 10 HPI as indicated by G3BP1 foci (Fig. 4a). For evaluating SG formation during infection, we focused on cells that expressed both G3BP1 foci (green puncta) and N (red). Examining WT SARS-CoV-2 at 10 HPI, we observed that cells with N staining lacked G3BP1 foci observed in non-infected bystander cells (Fig. 4b). In contrast, the YF/F17A mutant had many cells that were double positive for N and G3BP1 foci (Fig. 4c). Examining 24 HPI, we observed a similar trend, with mock-infected and bystander cells showing evidence of SG formation by G3BP1 foci (Fig. 4d through f). In WT infection at 24 HPI, cells expressing N had very low staining for G3BP1 foci and had evidence of cell fusion (Fig. 4e). In contrast, N and G3BP1 foci double-positive staining remained high in the YF/F17A mutant (Fig. 4f). We observed a significant increase in SG formation in the YF/F17A mutant compared to WT at both 10 and 24 HPI (Fig. 4g and h). We also noted that while N-positive cell numbers were equal at 10 HPI, WT-infected cells were twofold more abundant than the YF/F17A mutant at 24 HPI with or without arsenite treatment (Fig. 4i and j). Together, the results show that the YF/F17A mutant is unable to limit externally induced SG formation and has reduced infection over time.

**Fig 4:**
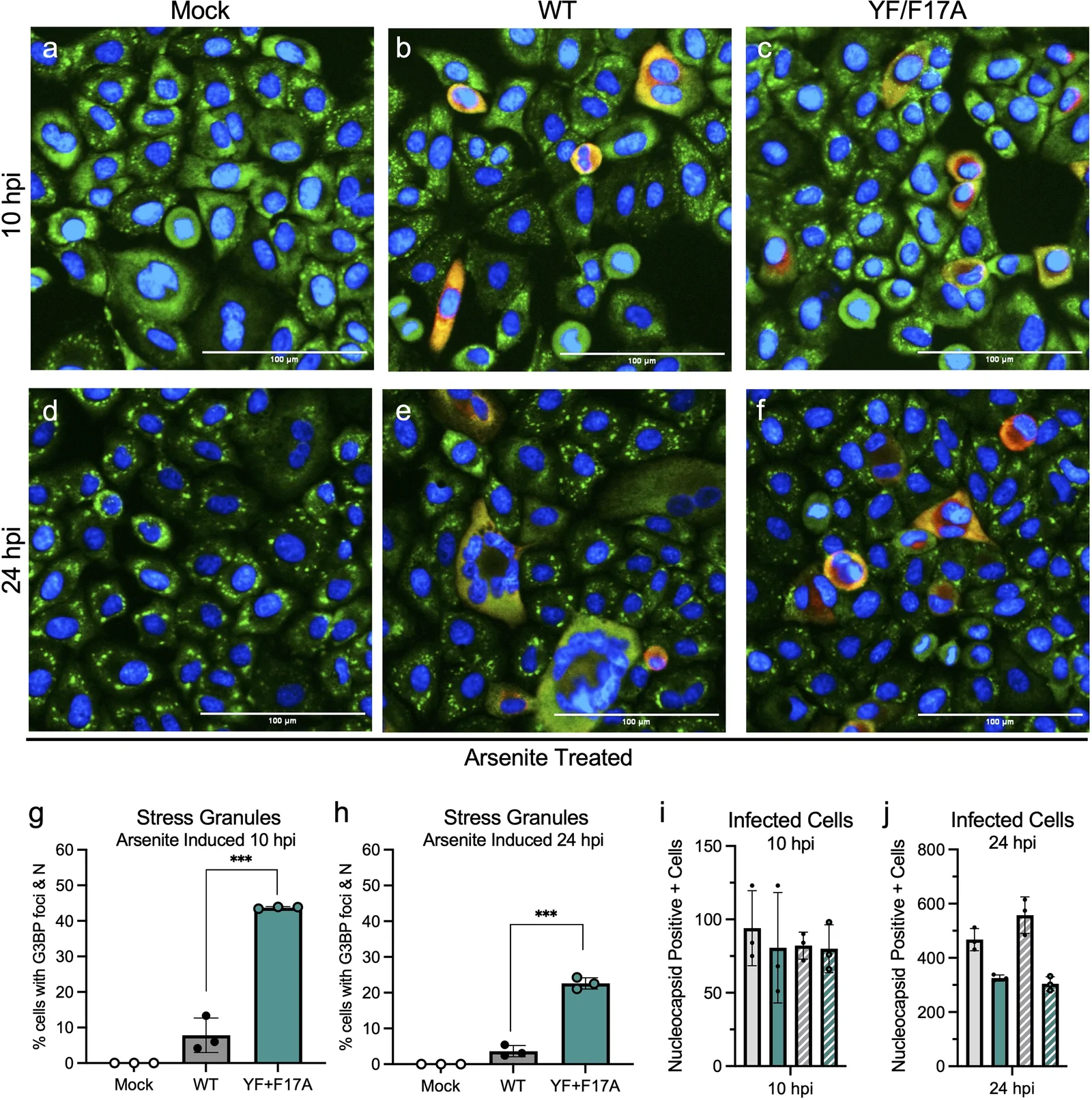
YF/F17A mutant is unable to control arsenite-induced stress granule formation. (**a–f**) A549-ACE2 cells were infected with mock PBS, WT, or YF/F17A mutant at an MOI of 2 and visualized for SG formation at (**a–c**) 10 HPI and (**d–f**) 24 HPI. G3BP1 (green), nucleocapsid (red), and nuclei (blue) were labeled to visualize by immunofluorescence imaging. (**g and h**) The total percentage of cells with G3BP1+ foci at (**g**) 10 HPI and (**h**) 24 HPI was calculated. (**i and j**) Total number of N-positive cells was calculated for WT (Gray) and YF/F17A mutant (teal) following control (solid) and arsenite treatment (hashed) at (**i**) 10 HPI and (**j**) 24 HPI. All quantitative imaging data were calculated using Fiji (ImageJ) software and are shown as mean ± SD. Statistical analysis was performed using a two-tailed Student’s *t*-test. ***P ≤ 0.001.

### YF/F17A mutant significantly attenuated weight loss and viral replication *in vivo*

Having established significant *in vitro* attenuation and robust induction of SGs, we next sought to evaluate the YF/F17A mutant *in vivo*. Using the Syrian golden hamster model for SARS-CoV-2 infection (43–46), we intranasally challenged animals with 10^5^ FFU of WT SARS-CoV-2, YF/F17A mutant, or PBS (mock) and monitored daily for weight loss and clinical signs of disease (Fig. 5a). At 2, 4, and 7 days post-infection (DPI), nasal washes were collected, followed by euthanasia and lung tissue harvested for measurement of viral titer, RNA, and histopathology. Consistent with prior studies, hamsters infected with SARS-CoV-2 WT had weight loss beginning at 2 DPI and continued until it peaked at ~15% at 6 DPI (Fig. 5b). In contrast, YF/F17A-infected hamsters were significantly attenuated in weight loss compared to WT infection throughout the time course. While mock-infected animals gained weight, the YF/F17A mutant-infected hamsters maintained their starting weight, showing no signs of disease. These data show that the YF/F17A double mutant is more attenuated than the individual NSP3 and N mutants (13, 14, 30). Overall, these results demonstrate that loss of SG antagonism by both NSP3 and N completely attenuated virus-induced weight loss in the SARS-CoV-2 hamster model of infection.

**Fig 5:**
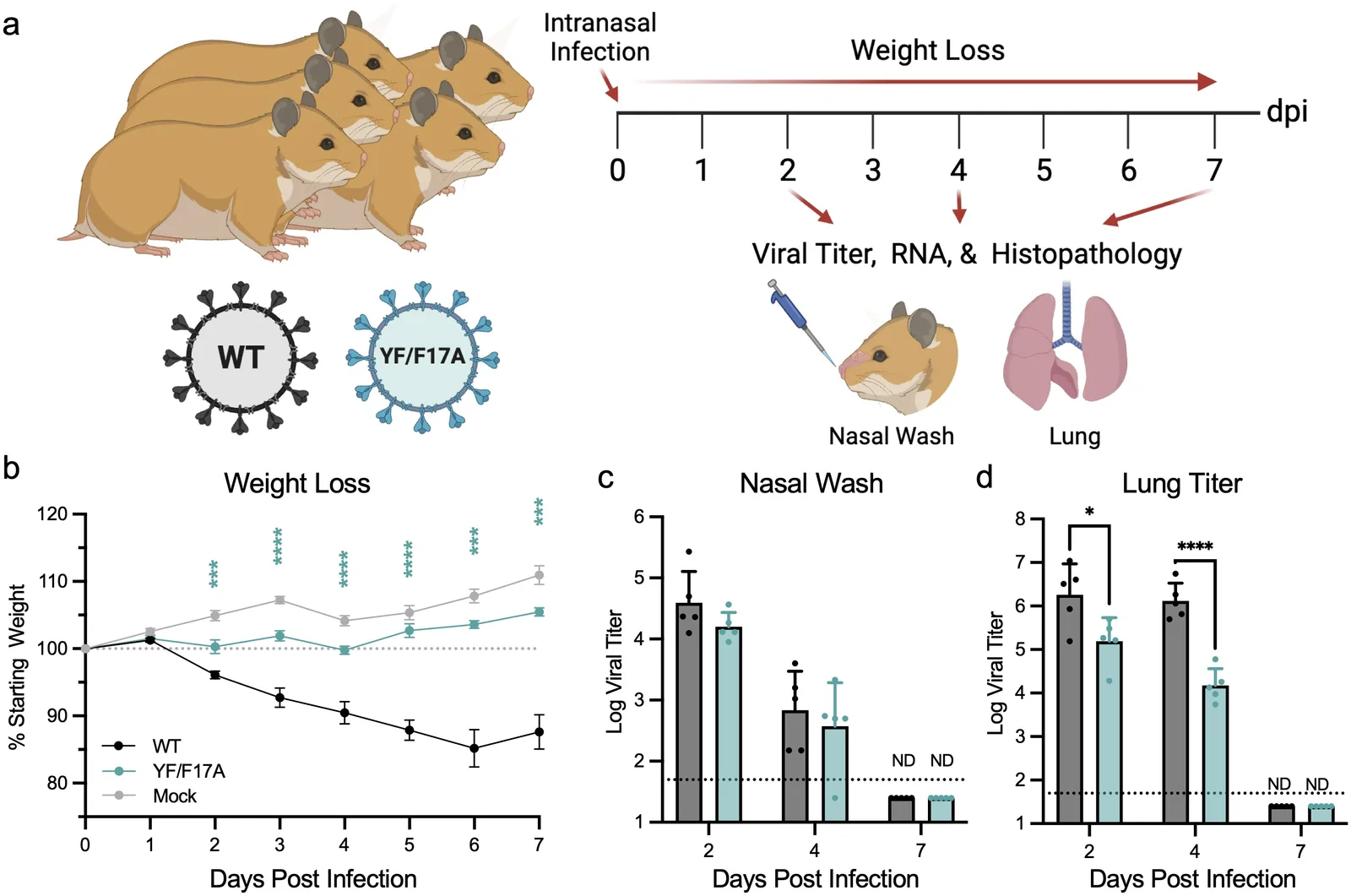
YF/F17A mutant has modest attenuation *in vivo*. (**a**) Schematic of golden Syrian hamster infection with WA-1 (WT) or YF/F17A mutant (teal) made using BioRender. (**b–d**) Three- to four-week-old male hamsters were intranasally inoculated with 10^5^ FFU of WT or YF and monitored daily for signs of disease and weight loss (**b**) over 7 days. Infectious titers were measured in the nasal wash (**c**) and lungs (**d**) on 2, 4, and 7 DPI. Each dot represents an infected animal. The dotted lines represent the assay limit of detection. Statistical significance was determined by a two-tailed Student’s *t*-test. *P ≤ 0.05; ***P ≤ 0.001; ****P < 0.0001. Panel a was created in BioRender. Menacherylab, T. (2026) https://BioRender.com/s645vmm.

Next, we examined viral replication in the upper and lower respiratory tract following infection with either WT or YF/F17A mutant. We observed no differences in nasal wash viral titers between WT and YF/F17A at 2 and 4 DPI (Fig. 5c); these results were consistent with prior studies that also observed no differences in the upper airway with the individual NSP3 and N mutants (14, 30). In contrast, we observed a consistent attenuation of the YF/F17A mutant in lung viral titers compared to WT (Fig. 5d). The initial 1-log reduction in YF/F17A at 2 DPI expanded to 2-logs at 4 DPI. This level of attenuation was more consistent than in both YF and F17A mutants individually (14, 30). In all animals, infection was resolved by 7 DPI in nasal washes and lung tissues. Together, these results show that disrupting the NSP3-FXR1 and N-G3BP1/2 binding interactions in combination drastically attenuates disease and viral load in lungs.

### YF/F17A mutant has reduced antigen staining in the lung

In addition to weight loss and replication, we also examined lung histopathology to evaluate changes between WT and the YF/F17A mutant. Staining for SARS-CoV-2 N revealed that viral antigen in the lung was reduced in the YF/F17A mutant relative to WT control (Fig. S1a through e). Beginning on day 2, we noted robust antigen staining in WT-infected hamster lungs compared to the YF/F17A-infected animals (Fig. S1a through c). The trend continued on day 4, with antigen largely absent in the YF/F17A group but maintained in the WT-infected group (Fig. S1c through e). By day 7, viral antigen had been cleared from both WT- and YF/F17A-infected animals. While we noted no significant shifts between WT and YF/F17A mutant in antigen distribution between airway and parenchyma (Fig. S1g through i), these overall results indicate that less antigen in the lungs of YF/F17A-infected hamsters corresponded with reduced lung viral titers (Fig. 5d).

### YF/F17A mutant has less lung immune infiltrate and inflammation

Examining immune infiltration and damage in the lung, we noted a significant attenuation in the YF/F17A mutant at late times post-infection. Using hematoxylin & eosin (H&E) staining, a board-certified pathologist evaluated lung sections in a blinded manner from days 2, 4, and 7 following WT and YF/F17A infection (Fig. S2a through f). While differences were noted in viral antigen staining, WT- and YF/F17A-infected hamsters had largely similar immune infiltration at both days 2 and 4 post-infection (Fig. S2a through d). For both WT and mutant infections, day 2 showed little evidence of immune infiltration compared to mock (Fig. S2a and b and g). By day 4, both infected groups showed increased immune infiltration and inflammation (Fig. S2c and d). However, on day 7, there was a delineation between the infected groups, with more severe immune infiltration in the WT-infected hamsters (Fig. S2e and h). In contrast, immune infiltration and damage plateaued at the day 4 level in the YF/F17A mutant and corresponded to reduced severity scores on day 7 (Fig. S2f and h). Together, the H&E data indicate that the YF/F17A mutant produced reduced inflammation and immune infiltration in the lung. These data are consistent with reduced disease in terms of weight loss (Fig. 5b). Overall, these results show clear attenuation of the YF/F17A mutant in terms of *in vivo* disease, which corresponded with the loss of SG control by the mutant virus.

### Host immune responses muted after YF/F17A mutant infection

We next evaluated the host responses in hamster lungs after infection with WT or YF/F17A. Total cellular RNA from infected lungs at day 4 was sequenced with Poly(A)-ClickSeq (47, 48) (Fig. 6). At 4 DPI, we observed 3,721 genes differentially expressed (*P*_adj_ <0.1) between WT and YF/F17A. Tracing the fold change compared to mock (Fig. 6a), most host genes show correlation between WT and YF/F17A; however, some show anti-correlated expression patterns (upregulated in WT vs downregulated in YF/F17A or vice versa). Examining gene ontology (GO) profiles, the YF/F17A mutant had reduced gene expression (*P*_adj_ <0.1 and fold change < −2) of several host immune response pathways compared to WT (Fig. 6b). These pathways included genes associated with innate immune responses, inflammation, and cytokine/chemokine. In contrast, YF/F17A had augmented expression of genes associated with RNA polymerase II and other cellular factors, although the significance values of these pathways were lower than the downregulation of immune pathways (Fig. 6c). These host pathways with modest *Z*-score increases in the mutant relative to WT SARS-CoV-2 were largely non-specific and suggested SG activity rather than host expression changes governed differential outcomes.

**Fig 6:**
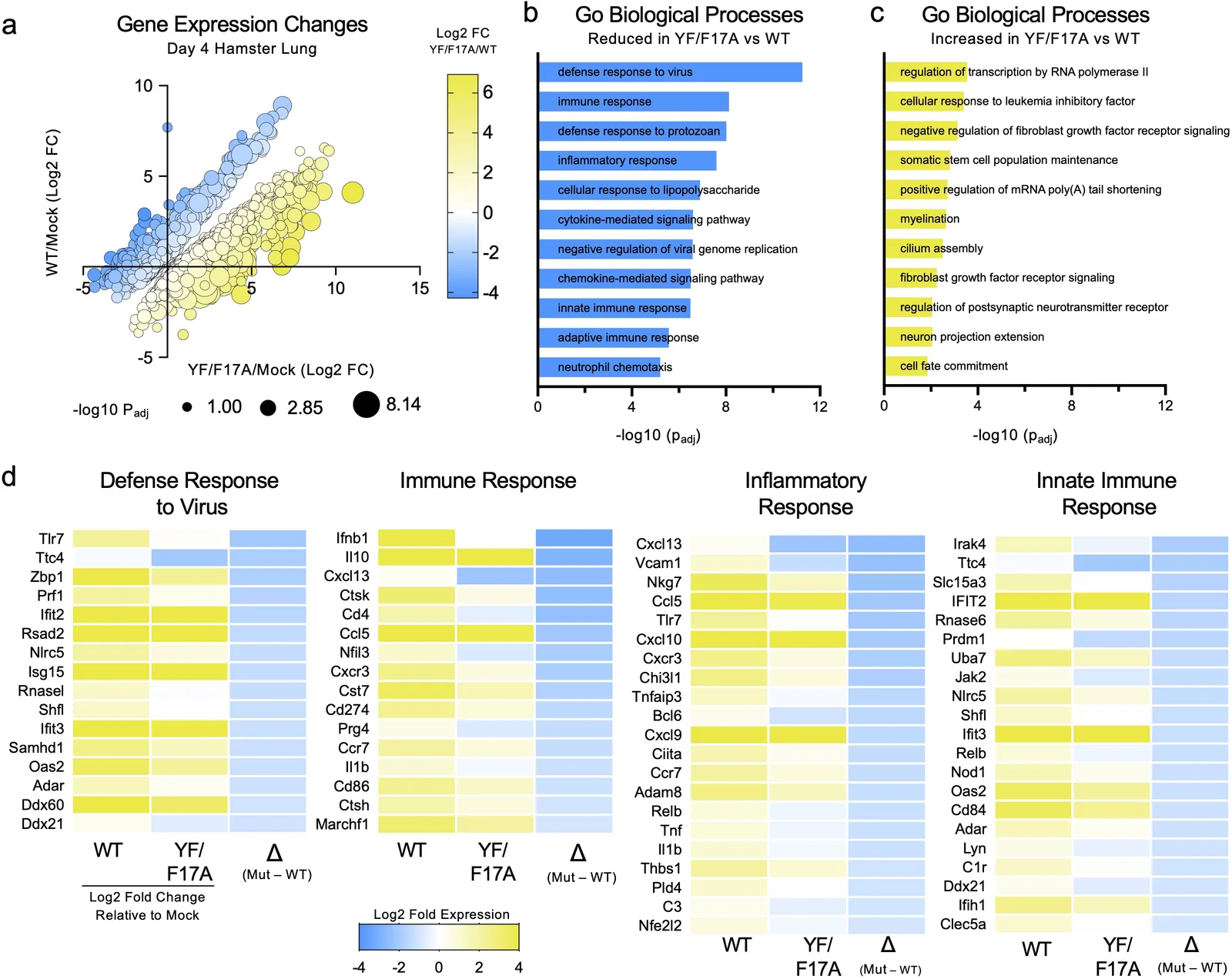
Host immune responses muted after YF/F17A mutant infection. (**a**) Host gene expression from day 4 hamster-infected lungs comparing WT (log2 fold changes vs mock, y-axis) to YF/F17A mutant (log2FC vs mock) shows distinct patterns of upregulation and downregulation. (**b and c**) Top GO terms discovered from significantly (*P*_adj_ < 0.1, |log2FC| > 1, *N* = 4 biological replicates) (**b**) downregulated or (**c**) upregulated host genes (WT vs YF/F17A) at day 4 post-infection. (**d**) Day 4 gene expression analysis of individual genes in selected GO terms associated with immune responses to infection. Log2 fold expression is depicted for WT and YF/F17A mutant relative to mock, as well as the ratio of YF/F17A to WT expression.

Focusing further on the GO, we examined RNA expression of genes related to innate immune responses: defense response to virus, immune response, inflammatory response, and innate immune response (Fig. 6d). For WT infection, in all four GO processes, we found upregulation of genes relative to mock-infected hamsters (1st column). While we observed some upregulation in the YF/F17A mutant relative to mock, these changes were muted or even inverse to what was observed in WT-infected hamsters (2nd column). Comparing mutant to WT directly, we observed that all the genes within these immune clusters had reduced expression in the YF/F17A mutant as compared to WT (3rd column). The results indicated that the YF/F17A mutant has a muted immune response and suggest that SG activation during CoV infection limits the need for innate immune activation. Together, the data confirm prior experiments showing that an augmented innate immune response is not responsible for attenuation of the YF/F17A mutant.

### YF/F17A mutant infection protects against SARS-CoV-2 rechallenge

The loss of NSP3 and N SG antagonism renders SARS-CoV-2 significantly attenuated both *in vitro* and *in vivo*. Notably, the YF/F17A mutant not only has reduced viral load but also diminished innate immune responses. This fact distinguishes it from other SARS-CoV-2 mutants that are inherently replication-deficient or induce exacerbated immune responses (49). It also leaves the possibility that reduced immune responses disrupt adaptive immunity, impacting protection from subsequent challenge. To explore this question, we determined whether the YF/F17A mutant could provide protection against subsequent rechallenge with WT SARS-CoV-2. Briefly, golden Syrian hamsters were initially challenged with 10^5^ FFU SARS-CoV-2 WT, YF/F17A mutant, or mock. The infected animals were monitored for weight loss and disease over a 7-day time course and had similar kinetics as in initial studies (Fig. S3). After 21 DPI, we rechallenged all groups with 10^5^ FFU of WT SARS-CoV-2 virus and monitored daily for weight loss and clinical signs of disease (Fig. 7a). At days 2, 4, and 7 post-rechallenge, serum, nasal wash, and lung specimens were collected for analysis (Fig. 7a). As expected, mock-vaccinated hamsters had significant weight loss following challenge with WT SARS-CoV-2 (Fig. 7b); these animals lost weight beginning at day 2 and we observed peak weight loss at day 6. In contrast, hamsters previously vaccinated with WT SARS-CoV-2 or the YF/F17A mutant had no weight loss following infection. The results indicate that despite reduced immune activation, infection with the YF/F17A mutant could protect against disease after rechallenge with WT SARS-CoV-2.

**Fig 7:**
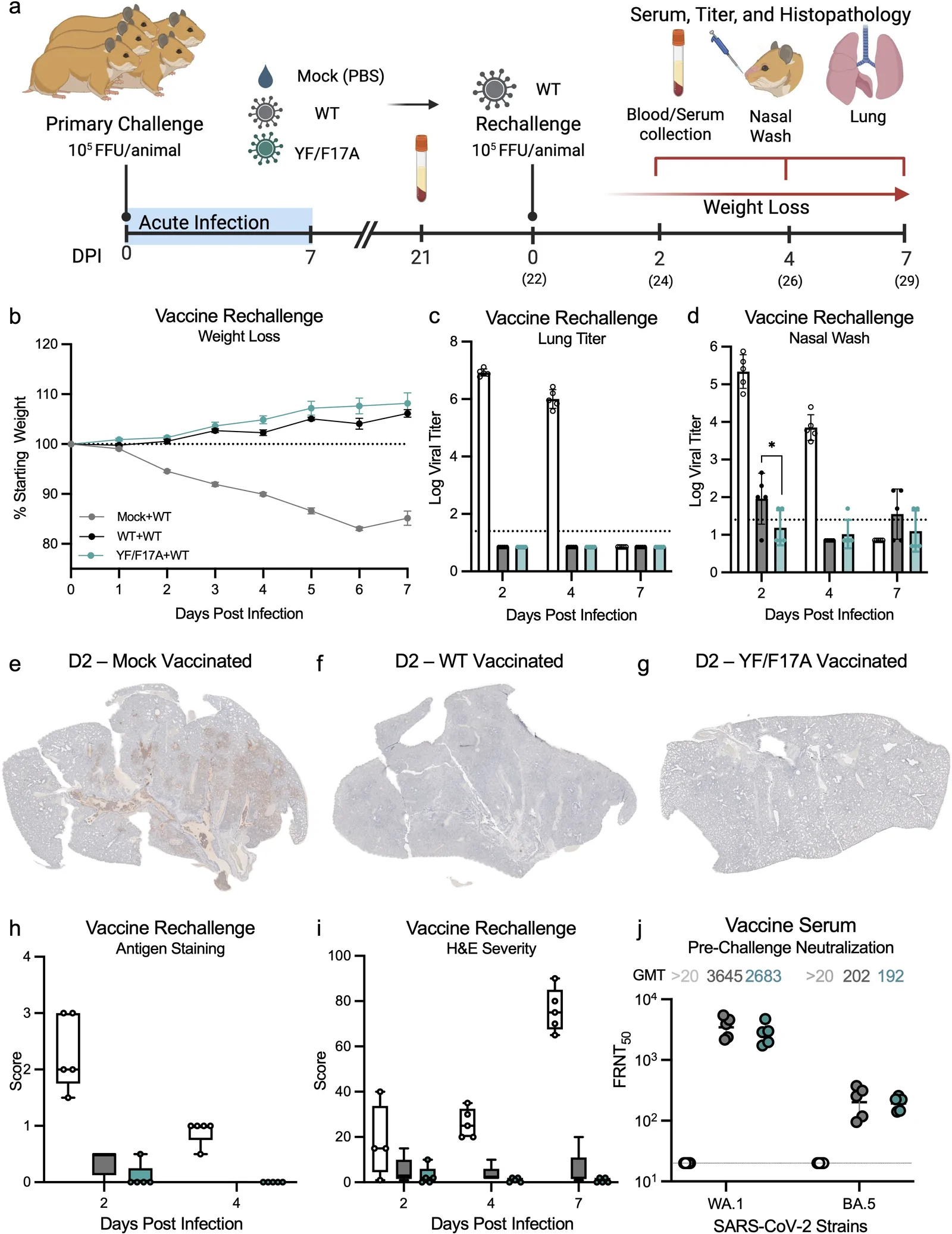
YF/F17A infection protects against rechallenge with SARS-CoV-2 WT. (**a**) Schematic of vaccination and rechallenge experiments. For primary challenge, 3- to 4-week-old male hamsters were intranasally vaccinated with 10^5^ FFU of WT (grey), YF/F17A mutant (teal), or mock (PBS, white), and monitored daily for disease and weight loss. At 21 days post-primary infection, hamsters were bled to collect serum and rechallenged the next day (22 DPI/D0) with 10^5^ FFU of WT. (**b–d**) Rechallenged animals were monitored daily for weight loss (**b**). Infectious titers were measured in the lung (**c**) and nasal wash (**d**) on days 2, 4, and 7 post-rechallenge. (**e–g**) Nucleocapsid antigen staining of left lung sections from (**e**) mock-vaccinated, (**f**) WT-vaccinated, or (**g**) YF/F17A mutant-vaccinated hamsters 2 days post-rechallenge. (**h**) Antigen staining was scored in a blinded manner for lung total score for mock-vaccinated (white), WT-vaccinated (grey), or YF/F17A-vaccinated (teal) infected sections at days 2 and 4 post-rechallenge. (**i**) H&E staining was scored in a blinded manner for total score for mock-vaccinated (white), WT-vaccinated (grey), or YF/F17A-vaccinated (teal) infected sections at days 2, 4, and 7 post-rechallenge. (**j**) Neutralizing antibody responses measured by live-virus-based focus reduction neutralization test (FRNT50) assays against SARS-CoV-2 WA1 and BA.5 using pre-challenge sera from mock-vaccinated (white), WT-vaccinated (grey), and YF/F17A-vaccinated (teal). FRNT50 geometric mean titers (GMTs) are indicated above each group. Each dot represents an experimental value from an infected animal. The dotted lines represent the assay limit of detection. Statistical significance was determined by a two-tailed Student’s *t*-test. *P ≤ 0.05. Panel a was created in BioRender. Menacherylab, T. (2026) https://BioRender.com/i44ze9p.

We then measured viral load in the upper and lower respiratory tracts of animals. Consistent with earlier studies (Fig. 5), mock-vaccinated animals had robust viral replication in both the lung and nasal wash after infection (Fig. 7c and d). Examining lung titer, the mock-vaccinated animals had significant viral loads at both day 2 and day 4 post-infection (Fig. 7c). In contrast, both WT- and YF/F17A-vaccinated animals had no detectable virus at either time point. Examining nasal wash, the mock-infected animals had robust titers at both day 2 and 4 post-infection (Fig. 7d). While titers were significantly diminished in the WT and YF/F17A mutant nasal wash samples, detectable virus was observed in both groups. These results are consistent with incomplete protection provided by infection/vaccination in the upper airway compared to control (50). Notably, we did observe a small but significant difference in day 2 nasal wash, as the WT had a slightly higher load than the YF/F17A mutant. Overall, the results indicate that despite the reduced immune induction during primary infection, the YF/F17A mutant limits viral replication to levels similar to vaccination with the WT SARS-CoV-2 strain.

We then examined lung histopathology from control and vaccinated hamsters (Fig. 7e through i; Fig. S4). Utilizing antigen staining for N, we found that mock-vaccinated hamsters had significant staining in both the airways and parenchyma following infection at both days 2 and 4 (Fig. 7e). In contrast, both WT- and YF/F17A-vaccinated animals showed minimal antigen staining in lung sections consistent with lung viral titer data (Fig. 7f through h). Examining immune pathology by H&E staining, we again observed robust inflammation and immune infiltrate in the mock-vaccinated hamsters infected with WT SARS-CoV-2 that progressed over the 7-day time course (Fig. 7i; Fig. S4a through c). In contrast, no significant immune infiltrate or inflammation was observed in the WT and YF/F17A group following rechallenge with WT SARS-CoV-2 (Fig. 7i; Fig. S4d through i). Together, the results indicate that protection against both viral replication and disease was achieved by YF/F17A mutant vaccination at levels similar to WT SARS-CoV-2 vaccination.

Finally, we examined serum neutralization from hamsters vaccinated with WT or YF/F17A mutant, or mock. Briefly, sera were collected from vaccinated and unvaccinated hamster groups 21 days post-vaccination and prior to rechallenge with SARS-CoV-2 WT (Fig. 7a). FRNT50 assays were performed as previously described (51) and demonstrated that both WT and YF/F17A mutant vaccination had similar neutralization of SARS-CoV-2 strains (Fig. 7j). Examining the backbone SARS-CoV-2 WA.1 strain, WT and YF/F17A mutant neutralizations were similar in their log GMT. Similarly, while neutralization against SARS-CoV-2 Omicron variant BA.5 had a 10-fold reduction compared to WA.1, both WT and YF/F17A vaccination resulted in nearly identical values. Together, the results indicate that the muted immune responses following primary infection with the YF/F17A mutant did not significantly impair vaccination efficacy. Overall, the serum findings combined with the challenge results indicate targeting SG antagonism has utility as a mechanism for live attenuated vaccines for SARS-CoV-2 and potentially other CoVs.

## DISCUSSION

SARS-CoV-2 devotes two viral proteins, NSP3 and N, to antagonize SG activity, suggesting a critical need to block this function. The YF/F17A mutant confirmed this reality, rendering the virus unable to replicate efficiently in cells (Fig. 1) or cause significant disease in hamsters (Fig. 5; Fig. S1 and S2). Importantly, the YF/F17A mutant attenuation was not associated with augmented innate immune responses. Cell culture studies indicated that innate immune responses were not augmented in the YF/F17A mutant (Fig. 2), and hamster lung RNA showed reduced activation of immune pathways (Fig. 6). Instead, attenuation of the YF/F17A mutant correlates with more activated SGs as measured by G3BP1 foci in infected cells (Fig. 3 and 4). Over time, this activation corresponded to fewer infected cells, indicating that SGs are likely responsible for the deficit in the YF/F17A mutant (Fig. 1 and 5). Overall, these results show that the YF/F17A mutant is more attenuated than the individual YF and F17A mutants alone. In addition, the data highlight the importance of disruption of SGs in SARS-CoV-2 infection and pathogenesis.

Both N and NSP3 are multifunctional proteins in the context of coronavirus infection with a variety of domains and roles during infection. During SARS-CoV-2 infection, the interplay between the two viral proteins allows kinetic control of SG formation. Early on, NSP3 uses its YF motif in its highly variable region to bind FMRP proteins and disrupt SG induction (14, 30). While less potent than N, NSP3 serves to delay SGs while N accumulates and performs other roles during the early parts of CoV infection. At late times (10 HPI), N functions to bind to G3BP1/2, master regulators of SGs, functionally disabling their activity during the latter part of infection (13, 16). In the absence of both SG antagonists, SARS-CoV-2 has attenuated replication and reduced pathogenesis that corresponds to accumulation of SGs in infected cells. Yet, SGs may only partially be responsible for the limitations of the YF/F17A mutant. Studies have indicated that while N binds to G3BP1 to disrupt SGs, it may also facilitate key aspects of viral replication via trafficking G3BP1 to the viral replication complex and aiding viral mRNA translation (18, 52). In this context, the hijacking of G3BP1 may have a dual role of being both pro-viral and antagonizing host SG responses. Coupled with the loss of early disruption by NSP3, the YF/F17A mutant is more limited by SG activation than each of the individual mutants (13, 14).

In addition to defining a critical need to control this pathway, our study also provides insights toward the mechanism of SG-mediated attenuation of SARS-CoV-2. The interplay between SGs and host innate immunity has led to mixed results in recent years. Some groups have found that SG activation amplifies innate immune responses (53–55). Other studies have argued that SGs modulate innate immune activation by acting as shock absorbers for overt activation (56). Still, other groups have reported no link between SGs and innate immune signaling (57, 58). While we do not explore this question in depth, our results indicate that loss of SG antagonism does not induce increased sensitivity to type I IFN pretreatment nor an augmented innate immune response to SARS-CoV-2 in cells (Fig. 2). Similarly, we find that interferon and inflammatory responses are reduced following *in vivo* infection with the YF/F17A mutant (Fig. 6). These results suggest SG activation may reduce innate immune activation through sequestration of viral RNA in SGs. However, the attenuated YF/F17A mutant replication may also limit IFN responses due to reduced viral load. Importantly, since CoVs possess numerous tools to prevent IFN induction, excessive IFN signaling is uncommon during infection. Thus, it is difficult to exclude a role for SGs in limiting excessive IFN signaling, which is not generally observed during CoV infection. Given these caveats, our results indicate that in the context of SARS-CoV-2 infection, loss of SG antagonism does not amplify innate immune responses and may reduce activation of innate immune responses.

Unlinking SG activity and innate immunity has potential implications for treating and preventing SARS-CoV-2 infection. Approaches to disrupt CoV infection for vaccination typically rely on reducing viral replication by targeting a key viral process or activating host immune responses via interferon pathways (59). Here, disrupting antagonism of SGs allows control of the SARS-CoV-2 infection through a distinct attenuation pathway independent of type I IFN. Using the YF/F17A mutant as a live attenuated vaccine, we found that animals infected with the mutant had minimal disease during acute infection (Fig. 5). Subsequent rechallenge with WT-SARS-CoV-2 demonstrated protection from both viral replication and disease at levels similar to WT vaccination (Fig. 7). Despite the reduced immune activation, the YF/F17A mutant produced similar neutralizing titers as WT and, coupled with robust protection from disease, indicated feasibility of this approach for live attenuated vaccination. Notably, the YF/F17A mutant offered a modest improvement in viral titer in the nasal wash, suggesting a potentially improved adaptive response (Fig. 7). Yet studies in hamsters are limited by lack of suitable immune reagents to fully characterize the B- and T-cell responses following both vaccinations. Overall, the results highlight targeting SG antagonism as a viable route of attenuation for SARS-CoV-2 live attenuated vaccines. However, SG-mediated attenuation of CoVs may be insufficient to be used as a live attenuated platform alone; thus, pairing this attenuation mechanism with replication defects, IFN sensitivity, or fidelity mutations may offer a tangible live attenuated vaccine approach moving forward. Since the molecular mechanisms governing SG antagonism are known for SARS-CoV-2, a combination approach targeting both NSP3 interaction with FMRP proteins and N interactions with G3BP1 may have utility as a drug treatment. In fact, studies targeting the N-G3BP1 interaction with drugs have shown some initial efficacy against SARS-CoV-2 (60, 61). Pairing this inhibition with NSP3/FMRP disruption may offer a novel, effective therapeutic pathway to activate SGs and inhibit SARS-CoV-2 infection.

Moving beyond SARS-CoV-2, these findings have implications for understanding the role of SGs in other coronaviruses. For N, examination of the N-terminal domain indicates conservation of F17 in similar locations of other CoVs, including SARS-CoV, implying the N-G3BP1 function may be broadly conserved within the Sarbecovirus family. In contrast, while the HVR residues are broadly conserved in the Sarbecovirus family, a similar motif is not found in the other CoV families (30). Importantly, other CoV families have developed other methods to disrupt SG functions directly. For example, MERS-CoV has been implicated in disrupting SG responses, with mutants lacking the ORF4a viral protein inducing more SGs (62). More broadly, CoVs employ several conserved viral proteins that disrupt double-stranded RNA production or immune recognition, including N, NSP1, NSP15, and NSP14 (13, 25, 27, 41, 63–70). With dsRNA acting as a stimulator of SG activation, disruptions of these viral antagonists have also been linked to SG activation during CoV infection (70–73). Together, these findings show that CoVs must spend significant genetic capital to modify and shape SG activation. As such, targeted approaches to disrupt this antagonism and activate SGs may be a viable approach for treatments and vaccine approaches across the entire CoV family.

Our findings also have implications for studying SGs in the context of viral infection. Many viruses utilize viral proteins to disrupt, delay, or inhibit SG functions, including influenza, alphaviruses, herpes viruses, and others. For decades, groups have utilized these viral families to explore the mechanisms and factors that contribute to SG activation and function. Among them, influenza NS1 has been consistently identified as a key SG antagonist, and numerous studies have utilized influenza NS1 mutants to identify key host factors that shape SG responses (74). Notably, influenza NS1 has several roles in viral infection, including antagonism of SG response and innate immunity (75). These overlapping roles and functions make interpretations of results more difficult, as numerous aspects of influenza infection in addition to SG activity are impacted. Here, we show that the YF/F17A mutant of SARS-CoV-2 maintains viral replication, lacks induction of innate pathways, and induces a significant increase in SGs contrasting to WT virus. With the recent downregulation to a lower biocontainment level, these results make the SARS-CoV-2 YF/F17A mutant a potentially useful tool to study and understand SG activity in the context of immunity to viral infection.

Overall, our study demonstrates the importance of controlling SG activity to SARS-CoV-2 infection and pathogenesis. Loss of NSP3 and N mechanisms to block SG formation results in significant attenuation of the SARS-CoV-2 mutant *in vitro* and *in vivo*. This attenuation corresponds to significant SG activation in the mutant and, notably, to reduced innate immune activation compared to WT SARS-CoV-2 following infection. The result is reduced viral replication and disease *in vivo*. Overall, the results highlight the importance of both N and NSP3 antagonism and highlight a novel pathway targeting SG antagonism as a means for CoV attenuation and treatment.

## MATERIALS AND METHODS

### Cell lines

African green monkey kidney Vero E6 cells were cultured in high-glucose Dulbecco’s modified Eagle medium (DMEM) (Gibco #11965-092) supplemented with 10% Fetal Clone II (FBS) (Hyclone; #SH30071.03) and 1% antibiotic/antimycotic (anti-anti) (Gibco #5240062). Vero E6 expressing human TMPRSS2 were grown in high-glucose DMEM supplemented with 10% FBS, 1% anti-anti, and 1 mg/mL Geneticin (G418) (Gibco; 10131035). Calu-3-2b4 (Calu-3) cells were grown in high-glucose DMEM with 10% defined FBS, 1% anti-anti, and 1 mg/mL sodium pyruvate (1 mM). A549 cells expressing human ACE2 were grown in 10% FBS, 1% anti-anti, and 10 µg/mL Blasticidin S HCl (Gibco; A1113902).

Vero E6 and Calu-3 cells were provided by Dr. Ralph Baric. A549-ACE2 cells were provided by Dr. Chien-Te Kent Tseng. All cell lines grow at 37°C with 5% CO_2_ and are mycoplasma-tested periodically (Last negative test in 2024). Single-cell FACS sorting (BD Aria Fusion) was applied to Vero-TMPRSS2 and A549-ACE2 cells to select for TMPRSS2- or ACE2-positive cell populations.

### Viruses

#### Reverse genetic clones

All recombinant viruses were generated using reverse genetics as previously described (31, 32). WT and mutant SARS-CoV-2 sequences are used from the USA-WA1/2020 isolate sequence provided by the World Reference Center for Emerging Viruses and Arboviruses (WRCEVA), originally obtained from the US Centers for Disease Control and Prevention (CDC) as previously described (34). The NSP3 YF mutant was constructed using standard cloning techniques as previously outlined for our reverse genetics system (31, 32), with virus stocks being amplified on Vero E6-TMPRSS2 to prevent mutations from occurring in the furin cleavage site (FCS) 33482650. All viruses were expanded once (P1) for use in studies and titered by focus-forming assay (FFA) and plaque assay (for plaque morphology). All infections and manipulations were performed in a biosafety level 3 (BSL-3) laboratory in accordance with approved protocols and with the use of appropriate personal protective equipment. P1 viral stocks were verified through Sanger sequencing of cDNA for mutations in the spike furin cleavage site, the NSP3-HVR, and N-F17A for the introduced mutations.

### Biosafety

The synthetic construction of SARS-CoV-2 NSP3 mutants was reviewed for DURC/P3CO policies and approved by the University of Texas Medical Branch Institutional Biosafety Committee. Procedures involving infectious SARS-CoV-2 were performed in the Galveston National Laboratory ABSL3 facility.

### *In vitro* infection

#### Viral replication kinetics

Viral replication studies were performed as previously described (30). Briefly, cells (Vero E6, A549-ACE2) were seeded in 6-well plates the day before infection. Calu-3 cells were seeded at high density and allowed to grow to confluence in 6-well plates before conducting the growth curve. Cells were infected at the indicated MOI for 45 min at 37°C with 5% CO_2_ and every 15 min. After absorption, plates were washed three times with PBS, and fresh medium was replaced in wells, indicating time zero. We sampled wells at specified time points. Three technical replicates were collected for each time point, and each experiment was performed twice. Samples were titrated with a focus-forming assay.

For high MOI infections, A549-ACE2 cells were plated in 12-well plates at 5 × 10^5^ cells per well. Viruses were diluted in PBS, added to cells, and incubated for 45 min at 37°C with 5% CO_2_ and rocked every 15 min. Cells were washed three times and replaced with complete DMEM (10% FBS with 1× anti-anti). For each time point, 200 μL of supernatant was collected for viral titers, and cells were lysed for qPCR gene expression analysis with TRIzol.

### Type I IFN assay

For IFN pretreatment, 100 units (U)/mL of recombinant IFN-β (PBL Laboratories) was added to Vero E6 cells 16–18 h prior to infection. Growth curves were conducted as mentioned above.

### Real-time quantitative PCR

#### RNA extraction and qPCR

Total RNA from cells was isolated with Direct-zol RNA Miniprep Plus kit (Zymo, #R2072) according to the manufacturer’s protocol. RNA was reverse transcribed to complementary DNA (cDNA) using the iScript cDNA synthesis kit according to the manufacturer’s instructions (Bio-Rad, 1708891). RT-qPCR was performed on cDNA using the Luna Universal qPCR Master Mix (NEB #M3003) per the manufacturer’s instructions. Fluorescent readings were measured on a Bio-Rad CFX Connect instrument using Bio-Rad CFX Maestro 1.1 (version 4.1.2433.1219). The primers used to amplify host targets were human GAPDH (forward: 5’- TCAAGATCATCAGCAATGCC; reverse: 5’- AAGTTGTCATGGATGACCTTGG), IFIT1 (forward: 5′-CCAAGGAGACCCCAGAAACC; reverse: 5′-CGCTACGTGGAGTGAGCTAG), IFIT3 (forward: 5′-AAGAACAAATCAGCCTGGTCAC; reverse: 5’- TCCCTTGAGACACTGTCTTCC), IFNβ (forward: 5′-AGTAGGCGACACTGTTCGTG; reverse: 5′-AGCCTCCCATTCAATTGCCA), OAS1 (forward: 5′-GAGCTCCTGACGGTCTATGC; reverse: 5′- TCATCGTCTGCACTGTTGCT), MDA5 (forward: 5′-AAGCCCACCATCTGATTGGAG; reverse: 5′-CCACTGTGGTAGCGATAAGCAG), PKR (forward: 5′-GAAGTGGACCTCTACGCTTTGG; reverse: 5′- GATGATGCCATCCCGTAGGTC). Average Ct values of technical replicates were normalized using the ΔΔCt method to the housekeeping gene (human GAPDH) and expressed as log_2_ relative fold change.

### Immunofluorescence and microscopy

A549-ACE2 cells were seeded on 96-well black-sided plates 1 day prior to infection. Infections were conducted at the indicated MOI in an inoculum volume of 50 µL per well. Incubation was conducted as mentioned in viral replication kinetics. Wells were replaced with fresh cell culture medium containing no selective antibiotics. To induce SG formation, wells were replaced with cell culture medium containing 1 mM sodium arsenite 30 min prior to the inactivation time point. Plates were inactivated in 10% buffered formalin at the indicated HPI. Antibody staining was adapted from reference 76. Briefly, fixed cells were permeabilized with 0.2% Triton X-100 in PBS for 10 min at room temperature (rt) and blocked with 2% BSA for 1 h. Samples were then incubated with primary antibodies: rabbit anti-G3BP1 (Cell signaling #61559) and mouse anti-nucleocapsid (Cell Signaling, 68344BC) overnight at 4°C. The next day, cells were washed three times with PBS and incubated with host-specific Alexa Fluor 488 (Invitrogen; A32731) and Alexa Fluor 555 (Invitrogen; A28180) secondary antibodies for 1 h at rt, away from light 39221199 (45). After secondary incubation, plates were washed three times, and nuclei were stained with 1 μg/mL Hoechst 3342 (Thermo Fisher, H1399) according to the manufacturer’s instructions for 15 min at rt. Images were captured using a Cytation 7 instrument at 20× magnification.

### Stress granule quantification

Quantification of SGs was adapted from a previous study (77). At least four areas were imaged in one well (repeated in triplicate) with consistent exposure settings for all images. Infected (N-positive) cells containing at least one granule were counted as SG+ using the multi-point tool in ImageJ 36534661 (46). The total number of cells expressing N was also counted using the multi-point tool in ImageJ. The percentage of stress granule-positive cells was then calculated by dividing the resulting number. For robust image analysis, standardized image acquisition practices were followed. Low to moderate cell densities were imaged to prevent negative impacts on cell counting and segmentation. Image acquisition was carried out using optimal constant exposure settings for each experiment. The “QuickFigures” plugin for ImageJ was used to prepare immunofluorescence panels (78).

### *In vivo* infection

#### Animal studies

Three-to-four-week male Syrian golden hamsters (HsdHAN:AURA strain) were purchased from Envigo, Indianapolis, IN, USA. For infection, hamsters were intranasally inoculated with 1 × 10^5^ FFU of SARS-CoV-2 WT or YF/F17A mutant in a 100 µL volume. Infected animals were weighed and monitored daily for illness over 7 days. Cohorts of five animals per group (including mock-infected animals) were anesthetized with isoflurane, and nasal washes were collected in 400 µL PBS on endpoint days (2, 4, and 7 DPI) for titers. Animals were then humanely euthanized by CO_2_ immediately following nasal washes for lung specimen collection. Lobes were stored in RNAlater solution (Invitrogen) for qPCR analysis and in PBS for titer analysis.

#### Rechallenge study

For rechallenge experiments, hamsters were intranasally inoculated with 1 × 10^5^ FFU of SARS-CoV-2 WT or YF/F17A mutant in a 100 µL volume PBS. Infected animals were weighed and monitored daily for illness over 7 days. Mock (PBS)-challenged groups were given 100 µL volume PBS. At day 21 post-primary challenge, serum was collected from all animals via retroorbital bleeding. Animals were allowed to recover and to challenge at day 22 post-primary challenge. Infected animals were weighed and monitored daily for illness over 7 days. Specimen collections were performed as previously described, with the addition of serum collection via cardiac puncture.

### Focus-forming assay

FFAs were performed as previously described with adaptations (79). Briefly, Vero E6 cells were seeded in 96-well black-walled plates to be 100% confluent the next day. Cell culture supernatants, nasal washes, or homogenized tissues containing SARS-CoV-2 underwent 10-fold serial dilutions in serum-free media. Twenty microliters of diluted and undiluted samples was added to wells and incubated for 45 min with rocking every 15 min. After absorption, methylcellulose overlay (0.85%) was added to each well, and cells were incubated at 37°C for 24 h. The next day, methylcellulose was removed, and cells were washed three times with PBS and fixed in 10% buffered formalin for 30 min at rt to inactivate virus. For staining, cells were permeabilized and blocked with PBS solution containing 0.1% saponin and 0.1% BSA (Perm. Buffer) for 30 min. Following incubation, α-SARS-CoV-2 nucleocapsid primary antibody (Cell Signaling, 68344) at 1:3,000 in Perm. buffer overnight at 4°C. Cells were washed three times with 1× DPBS before incubating with Alexa Fluor 555-conjugated α-mouse secondary antibody (Invitrogen, A28180) at 1:2,000 in Perm. Buffer for 1 h at rt wrapped in foil. Cells were washed three times with 1× DPBS, and fluorescent foci images were captured using a Cytation 7 cell imaging multi-mode reader (BioTek). Foci were counted using ImageJ free software (NIH).

#### Homogenization

Hamster lungs were infected as described in “Animal studies,” above, and the right inferior lobes were stored in sterile PBS, zirconia beads, and kept at −80°C. Tissues were homogenized using a MagNALyser (Roche Life Science) at 6,000 rpm for 1 min total.

### Histology

Left lung lobes were harvested from hamsters and fixed in 10% buffered formalin solution for at least 7 days. Fixed tissue was then embedded in paraffin, cut into 5 µm sections, and stained with H&E on a SAKURA VIP6 processor by the University of Texas Medical Branch Surgical Pathology Laboratory.

### Immunohistochemistry

Fixed and paraffin-embedded left lung lobes from hamsters were cut into 5 µm sections and mounted onto slides by the University of Texas Medical Branch Surgical Pathology Laboratory. Paraffin-embedded sections were warmed at 56°C for 10 min, deparaffinized with xylene (3 × 5-min washes) and graded ethanol (3× 100% 5-min washes, 1× 95% 5-min wash), and rehydrated in distilled water. After rehydration, antigen retrieval was performed by steaming slides in antigen retrieval solution (10 mM sodium citrate, 0.05% Tween-20, pH 6) for 40 min (boil antigen retrieval solution in a microwave, add slides to the boiling solution, and incubate in a steamer). After cooling and rinsing in distilled water, endogenous peroxidases were quenched by incubating slides in TBS with 0.3% H_2_O_2_ for 15 min, followed by 2 × 5-min washes in 0.05% TBST. Sections were blocked with 10% normal goat serum in BSA diluent (1% BSA in 0.05% TBST) for 30 min at room temperature. Sections were incubated with primary anti-N antibody (Sino #40143-R001) at 1:1,000 in BSA diluent overnight at 4°C (13, 80). Following overnight primary antibody incubation, sections were washed 3× for 5 min in TBST. Sections were incubated in secondary HRP-conjugated anti-rabbit antibody (Cell Signaling Technology #7074) at 1:200 in BSA diluent for 1 h at room temperature. Following secondary antibody incubation, sections were washed 3× for 5 min in TBST. To visualize antigen, sections were incubated in ImmPACT NovaRED (Vector Laboratories #SK-4805) for 3 min at room temperature before rinsing with TBST to stop the reaction, followed by 1× 5-min wash in distilled water. Sections were incubated in hematoxylin for 5 min at room temperature to counterstain before rinsing in water to stop the reaction. Sections were dehydrated by incubating in the previous xylene and graded ethanol baths in reverse order before mounting with coverslips.

### Next-generation sequencing libraries

For next-generation sequencing (NGS) analyses, RNA template was extracted from animal tissue with Direct-zol RNA miniprep kits (Zymo Research). To understand the transcriptomic changes of infected animals, extracted total cellular RNA from hamster lungs was subjected to PolyA-ClickSeq (47), which utilizes a semi-anchored oligo(dT) primer [5′- (T)_21_-3′] to specifically target polyA tails of cellular mRNA and a 1:5 azido-ddNTP:dNTP ratio to ensure sufficient termination of cDNA. The PolyA-ClickSeq library was constructed with a previously established protocol (81), and gel-selected libraries were single-end sequenced with ElementBio Aviti.

### Bioinformatics and data analysis

Raw reads sequenced from PolyA-ClickSeq libraries are processed and analyzed with a previously established *DPAC* (82) pipeline (https://github.com/andrewrouth/DPAC/) with parameters “*-p PMBCD,*” which detects and processes the polyA-containing reads and maps the upstream sequence of the polyA tail to the host reference. In this study, a PolyA-site clustering database was curated based on the published reference genome and annotation of *Mesocricetus auratus* (GenBank accession number GCA_000349665.1). Due to the incompleteness of the reference genome, the genome mapping criterion was slightly loosened in HISAT2 (83) stage with parameter “*--score-min L,0,-0.3.*” Differential gene expression profiles of mock-(PBS), WT-, and YF/F17A-infected hamster lung tissues were then analyzed with DESeq2 (84), which has been integrated into the *DPAC* pipeline (D stage) to reveal the normalized read count of each annotated gene.

For differential gene expression analyses, hierarchical gene clustering was conducted with *Cluster 3.0* (http://bonsai.hgc.jp/~mdehoon/software/cluster/). This is followed by TreeView (http://jtreeview.sourceforge.net/) to visualize the heat map and gene clusters. A gene is considered differentially expressed with Benjamini and Hochberg *P*_adj_ < 0.1 and |log2FC| > 0.585. Gene ontology analysis was conducted with DAVID (85) and differentially expressed genes (*P*_adj_ < 0.1, |log2FC| > 1) to highlight the most direct GO terms in biological process, cellular component, and molecular function. All plots and other statistics were conducted on GraphPad Prism (v.10.6.1).

### Focus reduction neutralization test

FRNT assays were performed as previously described (51, 86–88). Briefly, serum samples in duplicate were threefold diluted in eight serial dilutions using Dulbecco’s modified Eagle’s medium with an initial dilution of 1:10. Serially diluted samples were mixed with an equal volume of the desired SARS-CoV-2 variant (100–200 foci per well). The virus-serum mixtures were incubated at 37°C for 1 h in a round-bottom 96-well culture plate. After 1 h of incubation, the virus-serum mixture was added to VeroE6-TMPRSS2 cells and incubated at 37°C for an additional hour. Post-incubation, the mixture was removed from the cells, and 100 μL of prewarmed 0.85% methylcellulose overlay was added to each well. Plates were incubated at 37°C for 18–40 h (depending on variants). After the appropriate incubation time, the methylcellulose overlay was removed, and cells were washed with PBS and fixed with 2% paraformaldehyde for 30 min. After fixation, cells were washed twice with PBS and permeabilized using permeabilization buffer for at least 20 min. After permeabilization, cells were incubated with an anti–SARS-CoV-2 spike antigen primary antibody directly conjugated to Alexa Fluor 647 (CR3022-AF647) overnight at 4°C. Cells were then washed twice with 1× PBS and imaged on an ELISpot reader (CTL Analyzer).

## Data Availability

The raw data that support the findings of this study are available in the supplemental material. The raw sequencing data have been deposited in the NCBI BioProject database under accession no. PRJNA1477634.

## SUPPLEMENTAL MATERIAL

Data Set S1 jvi.00992-26-s0001.xlsx Source data for all main and supplemental figures.

Supplemental figures jvi.00992-26-s0002.pdf Fig. S1 to S4.

ASM does not own the copyrights to Supplemental Material that may be linked to, or accessed through, an article. The authors have granted ASM a non-exclusive, world-wide license to publish the Supplemental Material files. Please contact the corresponding author directly for reuse.
