# Supplementary material for: Antagonism of stress granules is key to SARS-CoV-2 infection and pathogenesis
Source: J Virol. 2026 Sep 8;100(10):e00992-26. doi: 10.1128/jvi.00992-26 (PMC13712807; doi:10.1128/jvi.00992-26)
Supplement: Supplementary file 1 [file jvi.00992-26-s0002.pdf]

Antagonism of stress granules key for SARS-CoV-2 infection and pathogenesis.
Alvarado et al.

Supplementary Figures

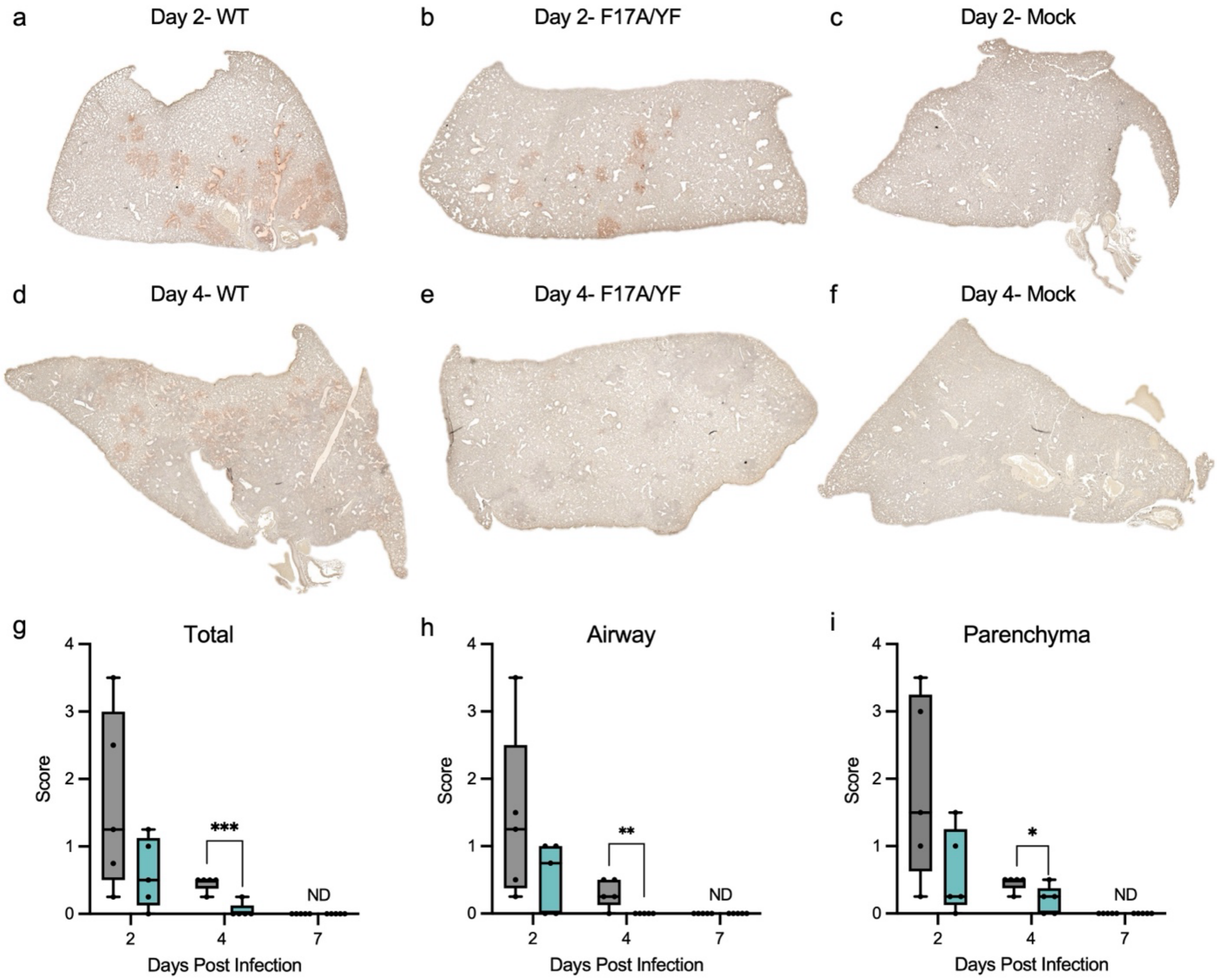

S. Figure 1: Viral antigen staining confirms YF/F17A attenuation *in vivo*.
(**A-F**) Nucleocapsid antigen staining of left lung section from hamsters infected with 10^5 FFU of either WT, YF/F17A mutant or mock PBS at (**A-C)** 2 or (**D-F**) 4 DPI. Antigen staining was scored in a blinded manner by location in the (**G**) lung total, (**H**) parenchyma, and (**I**) airway for WT (grey) or YF/F17A (teal) infected lungs. Statistical analysis was conducted using a two-tailed Student's *t* test. **P* ≤ 0.05; ***P* ≤ 0.01; and ****P* ≤ 0.001.

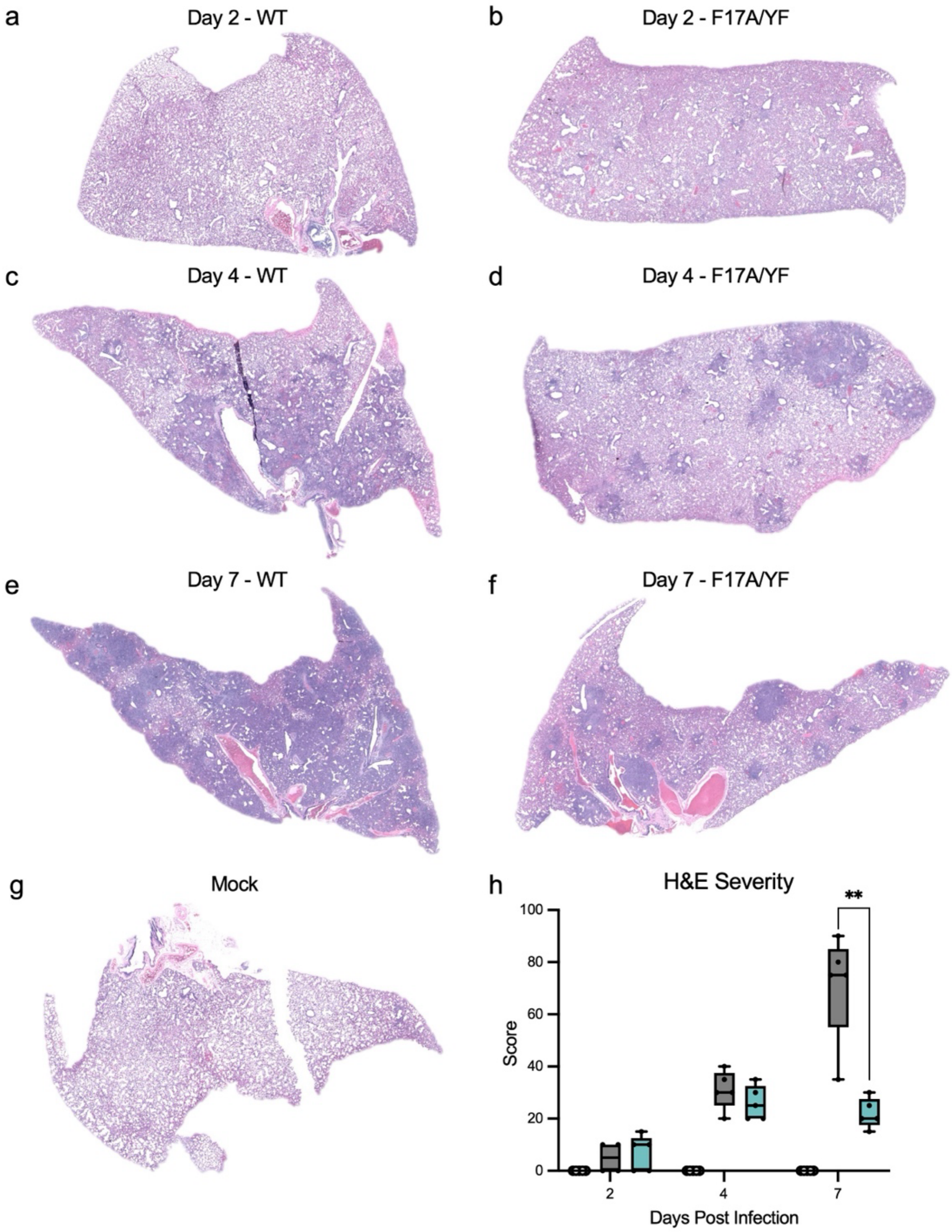

S. Figure 2: YF/F17A infection reveals reduced immune infiltration and damage.
Reduced inflammation and damage in YF/F17A infected lungs. (**A-G**) Representative H&E staining of the left lung of hamsters infected with 10^5 FFU of either WT or YF/F17A SARS-CoV-2 at (**A-B**) 2-, (**C-D**) 4-, or (**E-F**) 7 DPI or (**G**) mock. (**H**) Mock, WT (black), or YF/F17A (teal) lung sections from each day were scored for histopathological analysis, with sections from individual animals averaged and representing a single point. Statistical analysis was conducted using a two-tailed Student's t test. *$P \leq 0.05$; **$P \leq 0.01$; ***$P \leq 0.001$.

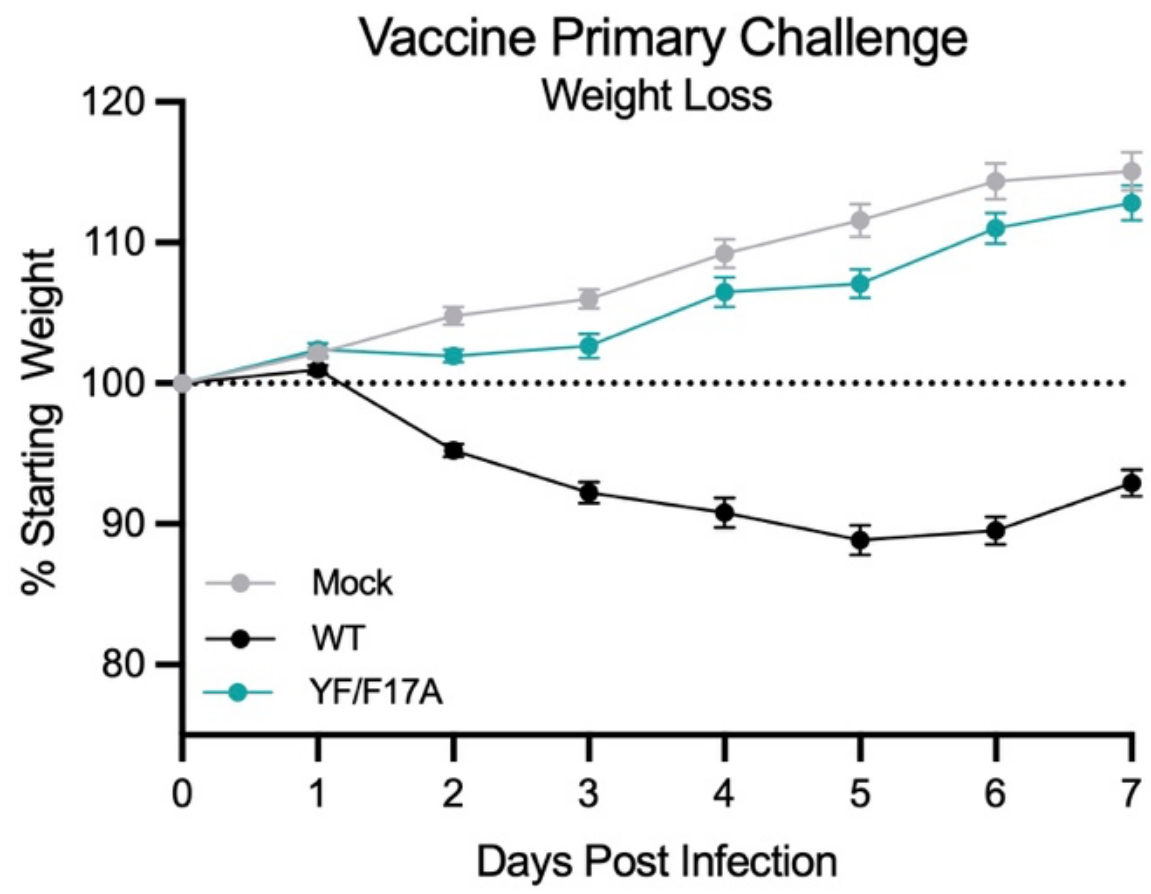

S. Figure 3: YF/F17A infection causes similar disease in vaccination as primary infection. For vaccination, three-to four-week-old male hamsters were intranasally inoculated with 10^5 FFU of WT, YF/F17A mutant, or equal volume PBS (mock), and monitored daily for signs of disease and weight loss over a 7-day time course.

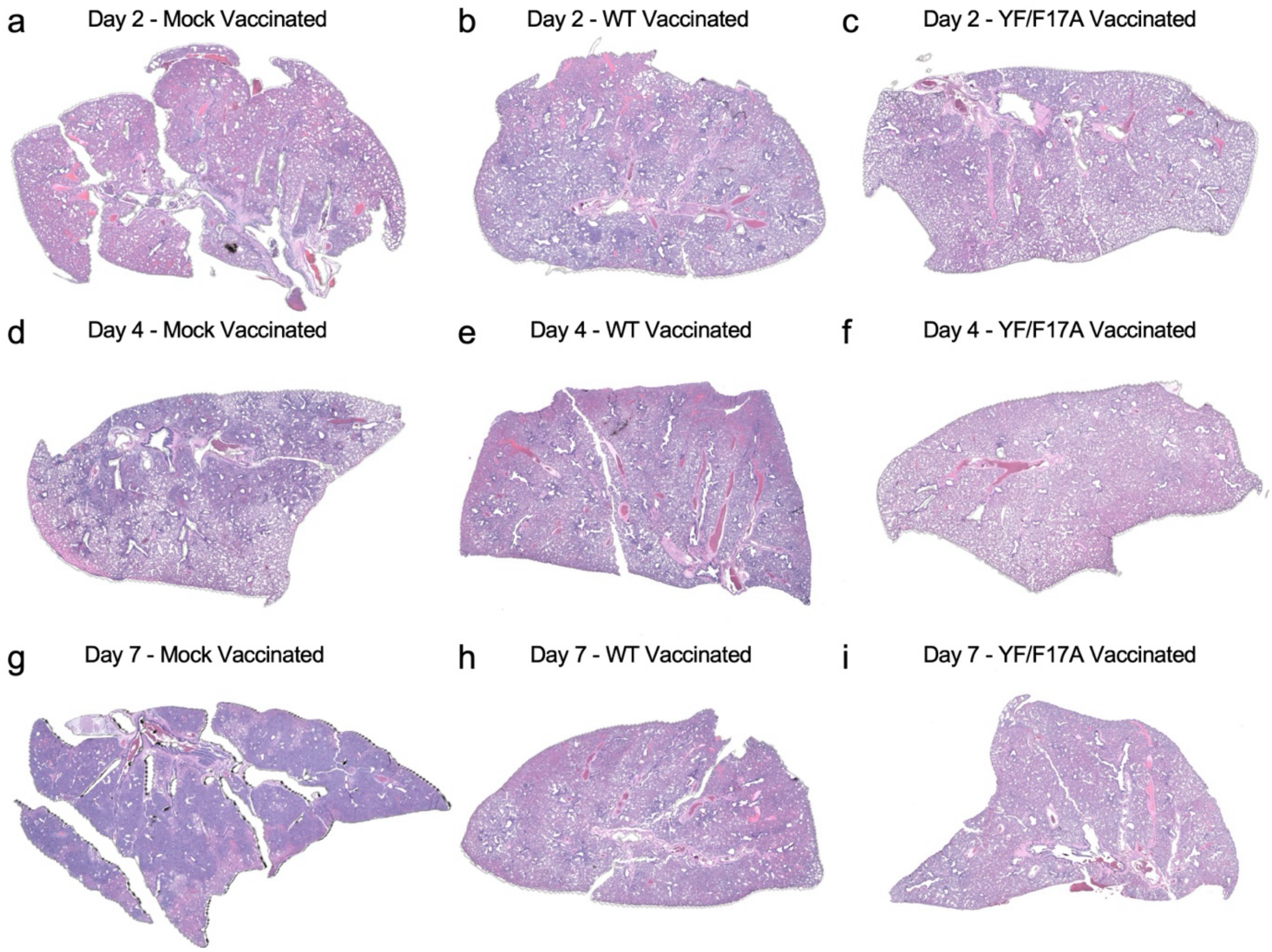

S. Figure 4: YF/F17A infection reveals reduced immune infiltration and damage.
Minimal inflammation or damage observed in vaccinated WT and YF/F17A infected hamster lungs. (**A-G**) Representative H&E staining of the left lung section of hamsters from mock vaccinated, WT-vaccinated or YF-F17A vaccinated rechallenged with 10^5 FFU of WT SARS-CoV-2 at (**A-C)** 2-, (**D-F**) 4-, or (**G-I**) 7 DPI or (G) mock.
